# Supplementary material for: Cancer stage at diagnosis by duration of pre-existing chronic analgesic use and anxiety or depression
Source: Nat Commun. 2025 Dec 16;17:7. doi: 10.1038/s41467-025-66334-2 (PMC12764921; doi:10.1038/s41467-025-66334-2)

Supplementary Information

Supplementary Table 1**: Grouping of drugs prescribed for patients with Chronic Analgesic Medication**

Supplementary Table 2**: Adjusted Odds Ratios (with 95% CIs) of advanced versus early-stage lung cancer and colon cancer diagnosis: socio-demographic variables, estimated using multivariable logistic regression analysis**

Supplementary Table 3**: Interactions between Chronic Analgesic Medication and age (Odds Ratios and 95% CI of advanced vs early stage at diagnosis), estimated using multivariable logistic regression analysis**

Supplementary Table 4**: Interactions between Anxiety / Depression and Chronic Analgesic Medication (Odds Ratios and 95% CI of advanced vs early stage at diagnosis), estimated using multivariable logistic regression analysis**

Supplementary Table 5**: Odds Ratios (with 95% CI) of advanced versus early stage among patients with Anxiety (+/- Depression) and Depression (+/- Anxiety), estimated using multivariable logistic regression analysis**

Supplementary Table 6**: Source of information on health conditions derived from primary care data using Cambridge Multimorbidity Score definitions**

Supplementary Table 7**: Socio-demographic characteristics of patients diagnosed with lung cancer between 2012-2018, by stage at diagnosis.** Pearson's Chi-squared test (two-sided), limiting to those with complete stage information, was used to test for an association between each listed characteristic and stage at diagnosis ($p= <0.05$).

Supplementary Table 8**: Socio-demographic characteristics of patients diagnosed with colon cancer between 2012-2018, by stage at diagnosis.** Pearson's Chi-squared test (two-sided), limiting to those with complete stage information, was used to test for an association between each listed characteristic and stage at diagnosis ($p= <0.05$).

Supplementary Figure 1**: Patients included in lung and colon cancer patient cohorts**

Supplementary Figure 2**: Distribution of stage at diagnosis (%) by presence of Anxiety / Depression or Chronic Analgesic Medication. a** Lung cancer patients (n=6,828) **b**. Colon cancer patients (n=4,194)

Supplementary Figure 3**: Type of drugs prescribed to patients with Chronic Analgesic Medication use, by cancer and by recent-onset or persistent Chronic Analgesic Medication use. a.** Colon cancer patients (recent-onset use, n=575; persistent use, n=427) **b.** Lung cancer patients (recent-onset use,n=1,859; persistent use, n=600)

Supplementary Figure 4**: Prevalence (% of patients) of Anxiety versus Depression among cancer patients, according to duration. a** Lung cancer patients (n=6,828) **b**. Colon cancer patients (n=4,194)

Supplementary Figure 5**: Directed Acyclic Graph showing associations between Chronic Analgesic Medication or Anxiety / Depression, covariates and stage at diagnosis**

Supplementary Table 1: Grouping of drugs prescribed for patients with Chronic Analgesic Medication

Group	Drug name
Opioid	Buprenorphine
	Buprenorphine hydrochloride
	Codeine phosphate
	Cyclizine hydrochloride / Dipipanone hydrochloride
	Dextropropoxyphene hydrochloride
	Diamorphine hydrochloride
	Dihydrocodeine tartrate
	Fentanyl
	Meptazinol hydrochloride
	Morphine sulfate
	Naloxone hydrochloride / Oxycodone hydrochloride
	Oxycodone hydrochloride
	Oxycodone hydrochloride / Naloxone hydrochloride
	Pethidine hydrochloride
	Tapentadol hydrochloride
	Tramadol hydrochloride
Non-steroidal anti-inflammatory drugs (NSAID)	Aceclofenac
	Celecoxib
	Diclofenac
	Etodolac
	Etoricoxib
	Fenbufen
	Flurbiprofen
	Indometacin
	Ketoprofen
	Ketoprofen / Omeprazole
	Mefenamic acid
	Meloxicam
	Nabumetone
	Naproxen
	Piroxicam
Neuropathic	Carbamazepine
	Gabapentin
	Lacosamide
	Lamotrigine
	Levetiracetam
	Oxcarbazepine
	Phenytoin sodium
	Pregabalin
	Sodium valproate
	Topiramate
Other	Clobazam
	Clonazepam
	Nefopam hydrochloride
	Phenobarbital
	Primidone
	Racemic camphor / Capsicum oleoresin / Menthol / Methyl salicylate
Simple analgesia	Ibuprofen
	Paracetamol

Supplementary Table 2: Adjusted† Odds Ratios (with 95% CIs) of advanced versus early-stage lung cancer and colon cancer diagnosis: socio-demographic variables, estimated using multivariable logistic regression analysis

	**Lung cancer**	**Colon cancer**
	OR (95% CI)	
**Age at diagnosis**		
<60 years	0.97 (0.78, 1.20)	1.07 (0.87, 1.33)
60-69 years (Ref.)	1.00	1.00
70-79 years	0.85 (0.72, 0.99)	0.94 (0.78, 1.13)
80+ years	0.91 (0.76, 1.09)	0.88 (0.73, 1.07)
**Sex**		
Male (Ref.)	1.00	1.00
Female	0.83 (0.73, 0.94)	0.98 (0.86, 1.13)
**Deprivation quintile**		
Least deprived (Ref.)	1.00	1.00
2	0.97 (0.79, 1.19)	0.85 (0.70, 1.03)
3	0.97 (0.79, 1.18)	0.90 (0.74, 1.10)
4	0.99 (0.81, 1.21)	1.02 (0.82, 1.27)
Most deprived	1.12 (0.92, 1.36)	0.94 (0.75, 1.18)

†Models adjusted for Anxiety / Depression, Chronic Analgesic Medication use, age, sex, IMD income quintile, clinical and healthcare covariates (additional results from these models are reported in Table 1).

Supplementary Table 3: Interactions between Chronic Analgesic Medication and age (Odds Ratios and 95% CI of advanced vs early stage at diagnosis), estimated using multivariable logistic regression analysis

		Main effects only	
		Lung cancer	Colon cancer
		**OR (95% CI)**	
**Chronic Analgesic Medication**	None (Ref.)	1.00	1.00
	Recent-onset	**1.68 (1.44, 1.96)**	**2.24 (1.81, 2.78)**
	Persistent or Historic	**0.37 (0.31, 0.44)**	0.91 (0.74, 1.11)
**Age at diagnosis**	<60 Years	0.96 (0.77, 1.18)	1.06 (0.86, 1.31)
	60-69 years	1.00	1.00
	70-79 years	0.85 (0.73, 1.00)	0.95 (0.79, 1.14)
	80+ years	0.93 (0.78, 1.10)	0.90 (0.74, 1.09)

		With interaction term	
		Lung cancer	Colon cancer
**Chronic Analgesic Medication**	**Age at diagnosis**	**OR (95% CI)**	
None (Ref.)	<60 years	1.01 (0.76, 1.34)	0.91 (0.71, 1.17)
	60-69 years (Ref.)	1.00	1.00
	70-79 years	0.99 (0.81, 1.21)	1.01 (0.82, 1.26)
	80+ years	**1.28 (1.03, 1.60)**	1.04 (0.83, 1.31)
Recent-onset	<60 Years	**2.09 (1.41, 3.10)**	**4.63 (2.51, 8.55)**
	60-69 years	**2.60 (1.89, 3.57)**	**1.74 (1.12, 2.71)**
	70-79 years	**1.65 (1.26, 2.15)**	**2.68 (1.76, 4.07)**
	80+ years	1.31 (0.98, 1.76)	**1.71 (1.19, 2.46)**
Persistent or Historic	<60 Years	**0.45 (0.30, 0.66)**	**1.57 (1.01, 2.43)**
	60-69 years	**0.49 (0.36, 0.67)**	1.27 (0.88, 1.83)
	70-79 years	**0.37 (0.27, 0.51)**	0.73 (0.53, 1.02)
	80+ years	**0.27 (0.18, 0.40)**	**0.59 (0.40, 0.89)**

Abbreviations: CI, Confidence Intervals

Supplementary Table 4: Interactions between Anxiety / Depression and Chronic Analgesic Medication (Odds Ratios and 95% CI of advanced vs early stage at diagnosis), estimated using multivariable logistic regression analysis

	Main effects only	
	**Lung cancer**	**Colon Cancer**
	**OR (95% CI)**	
**Anxiety or Depression**		
None	1.00	1.00
Recent-onset	1.19 (0.94, 1.51)	1.02 (0.71, 1.46)
Persistent or historic	**0.83 (0.70, 0.98)**	**0.77 (0.64, 0.93)**
**Chronic Analgesic Medication**		
None	1.00	1.00
Recent-onset	**1.63 (1.39, 1.91)**	**2.25 (1.81, 2.79)**
Persistent or historic	**0.39 (0.32, 0.47)**	0.96 (0.78, 1.18)

**Anxiety or Depression**	**Chronic Analgesic Medication**	*With interaction term* **OR (95% CI)**	
None	None	1.00	1.00
None	Recent-onset	**1.72 (1.43, 2.08)**	**2.45 (1.90, 3.17)**
None	Persistent or historic	**0.36 (0.28, 0.46)**	1.04 (0.82, 1.33)
Recent-onset	None	1.19 (0.80, 1.77)	1.09 (0.62, 1.93)
Recent-onset	Recent-onset	**1.74 (1.29, 2.36)**	**2.21 (1.31, 3.73)**
Recent-onset	Persistent or historic	0.77 (0.40, 1.50)	1.02 (0.46, 2.24)
Persistent or historic	None	0.84 (0.67, 1.06)	0.90 (0.70, 1.15)
Persistent or historic	Recent-onset	1.26 (0.90, 1.77)	1.42 (0.88, 2.28)
Persistent or historic	Persistent or historic	**0.33 (0.26, 0.42)**	**0.67 (0.50, 0.91)**

Abbreviations: CI, Confidence Intervals

Supplementary Table 5: Odds Ratios (with 95% CI) of advanced versus early stage among patients with Anxiety (+/- Depression) and Depression (+/- Anxiety), estimated using multivariable logistic regression analysis

	**Lung cancer**	**Colon cancer**
	OR* (95% CI)	
**Anxiety (including patients who also had Depression)**		
None (Ref.)	1.00	1.00
Recent	**1.45 (1.07, 1.99)**	1.33 (0.84, 2.11)
Persistent or Historic	1.07 (0.85, 1.36)	1.06 (0.81, 1.38)
**Anxiety (excluding patients who also had Depression)**		
None (Ref.)	1.00	1.00
Recent	**1.99 (1.23, 3.19)**	**2.13 (1.08, 4.19)**
Persistent or Historic	1.39 (0.95, 2.02)	1.20 (0.83, 1.74)
**Depression (including patients who also had Anxiety)**		
None (Ref.)	1.00	1.00
Recent	0.95 (0.73, 1.24)	0.88 (0.59, 1.29)
Persistent or Historic	**0.72 (0.60, 0.86)**	**0.63 (0.51, 0.78)**
**Depression (excluding patients who also had Anxiety)**		
None (Ref.)	1.00	1.00
Recent	1.00 (0.74, 1.35)	0.68 (0.43, 1.07)
Persistent or Historic	**0.69 (0.56, 0.85)**	**0.63 (0.49, 0.80)**

* Models adjusted for age, sex, deprivation quintile, Chronic Analgesic Medication, number of GP visits, presence of site-specific and general cancer symptoms and total number of physical morbidities
Abbreviations: CI, Confidence Intervals

Supplementary Table 6: Source of information on health conditions derived from primary care data using Cambridge Morbidity Score definitions

Health condition	Source of data to define condition
Alcohol problems	Read code recorded
Anorexia or bulimia	Read code recorded
Anxiety / Depression	Read code recorded OR ≥4 anxiolytic / hypnotic prescriptions in a 12-month period
Asthma	Read code recorded AND any (related) prescription
Atrial fibrillation	Read code recorded
Blindness	Read code recorded
Bronchiectasis	Read code recorded
Congestive Heart Disease	Read code recorded
Chronic Analgesic Medication	≥4 prescription-only analgesics OR ≥4 specified anti-epileptics in a 12-month period, in the absence of epilepsy
Chronic Kidney Disease	Read code recorded
Chronic Liver Disease	Read code recorded
Chronic Obstructive Pulmonary Disease	Read code recorded
Dementia	Read code recorded
Diabetes	Read code recorded
Diverticular Disease of the Intestine	Read code recorded
Epilepsy	Read code recorded AND any anti-epileptic prescription
Heart failure	Read code recorded
Hearing loss	Read code recorded
Hypertension	Read code recorded
Inflammatory Bowel Disease	Read code recorded
Irritable Bowel Syndrome	Read code recorded OR ≥4 prescription-only antispasmodic medication in a 12-month period
Learning difficulty	Read code recorded
Migraine	≥4 prescription-only medicine anti-migraine prescriptions in 12-month period
Multiple sclerosis	Read code recorded
Parkinsons Disease	Read code recorded
Peptic ulcer	Read code recorded
Prostate disorders	Read code recorded
Psoriasis or eczema	Read code recorded AND ≥4 related prescriptions (excluding emollients) in 12-month period
Psychoactive substance misuse	Read code recorded
Peripheral Vascular Disease	Read code recorded
Rheumatoid arthritis	Read code recorded
Schizophrenia	Read code recorded OR Lithium prescription ever recorded
Sinus condition	Read code recorded
Stroke & transient ischaemic attack	Read code recorded
Thyroid disorders	Read code recorded

Supplementary Table 7: Socio-demographic characteristics of patients diagnosed with lung cancer between 2012-2018, by stage at diagnosis. Pearson's Chi-squared test (two-sided), limiting to those with complete stage information, was used to test for an association between each listed characteristic and stage at diagnosis (p= <0.05).

	Stage at diagnosis														
	Stage I		Stage II		Stage III		Stage IV		Early (Stages I or II)	Advanced (Stages III or IV)	Missing			All patients	
Lung cancer patients	n	%	n	%	n	%	n	%	%	%	n	%	p-value	N	%
**Age at diagnosis (years)**													0.02		
24-44	14	1.5	1	0.2	8	0.6	26	0.8	1.0	0.7	9	1.3		58	0.8
45-59	109	11.4	55	11.4	197	15.1	436	13.0	11.4	13.6	37	5.2		834	12.2
60-69	258	26.9	124	25.7	389	29.8	955	28.4	26.5	28.8	126	17.6		1,852	27.1
70-79	352	36.7	170	35.3	455	34.8	1,077	32.0	36.2	32.8	207	28.9		2,261	33.1
80+	226	23.6	132	27.4	257	19.7	871	25.9	24.8	24.1	337	47.1		1,823	26.7
**Sex**													<0.01		
Male	402	41.9	264	54.8	713	54.6	1,716	51.0	46.2	52.0	349	48.7		3,444	50.4
Female	557	58.1	218	45.2	593	45.4	1,649	49.0	53.8	48.0	367	51.3		3,384	49.6
**Deprivation quintile (IMD income domain)**													0.75		
1 (Least deprived)	158	16.5	95	19.7	204	15.6	605	18.0	17.6	17.3	147	20.5		1,209	17.7
2	182	19.0	81	16.8	240	18.4	599	17.8	18.3	18.0	128	17.9		1,230	18.0
3	197	20.5	100	20.7	268	20.5	671	19.9	20.6	20.1	143	20.0		1,379	20.2
4	214	22.3	109	22.6	286	21.9	729	21.7	22.4	21.7	136	19.0		1,474	21.6
5 (Most deprived)	208	21.7	97	20.1	308	23.6	761	22.6	21.2	22.9	162	22.6		1,536	22.5
**Total by stage**	959	14	482	7.1	1306	19.1	3,365	49.3	21.1	68.4	716	10.5		6,828	100.0

Supplementary Table 8: Socio-demographic characteristics of patients diagnosed with colon cancer between 2012-2018, by stage at diagnosis. Pearson's Chi-squared test (two-sided), limiting to those with complete stage information, was used to test for an association between each listed characteristic and stage at diagnosis (p= <0.05).

	Stage at diagnosis														
	Stage I		Stage II		Stage III		Stage IV		Early (Stages I or II)	Advanced (Stages III or IV)	Missing			All patients	
Colon cancer patients	n	%	n	%	n	%	n	%	%	%	n	%	p-value	N	%
**Age at diagnosis (years)**													0.10		
26-44	15	3.0	33	3.0	27	2.8	29	2.7	3.0	2.8	15	2.6		119	2.8
45-59	73	14.4	140	12.9	147	15.3	187	17.4	13.4	16.4	57	10.0		604	14.4
60-69	146	28.8	238	21.9	247	25.7	257	24.0	24.1	24.8	75	13.2		963	23.0
70-79	156	30.8	340	31.3	296	30.8	302	28.1	31.1	29.4	128	22.5		1,222	29.1
80+	117	23.1	335	30.8	243	25.3	298	27.8	28.4	26.6	293	51.6		1,286	30.7
**Sex**													0.89		
Male	261	51.5	536	49.4	490	51.0	532	49.6	50.0	50.3	249	43.8		2,068	49.3
Female	246	48.5	550	50.6	470	49.0	541	50.4	50.0	49.7	319	56.2		2,126	50.7
**Deprivation quintile (IMD income domain)**													0.32		
1 (Least deprived)	131	25.8	240	22.1	231	24.1	268	25.0	23.3	24.5	159	28.0		1,029	24.5
2	120	23.7	272	25.0	221	23.0	231	21.5	24.6	22.2	139	24.5		983	23.4
3	106	20.9	263	24.2	219	22.8	243	22.6	23.2	22.7	96	16.9		927	22.1
4	76	15.0	163	15.0	161	16.8	181	16.9	15.0	16.8	94	16.5		675	16.1
5 (Most deprived)	74	14.6	148	13.6	128	13.3	150	14.0	13.9	13.7	80	14.1		580	13.8
**Total by stage**	507	12.1	1,086	25.9	960	22.9	1,073	25.6	38.0	48.5	568	13.5		4,194	100.0

Supplementary Figure 1: Patients included in lung and colon cancer patient cohorts

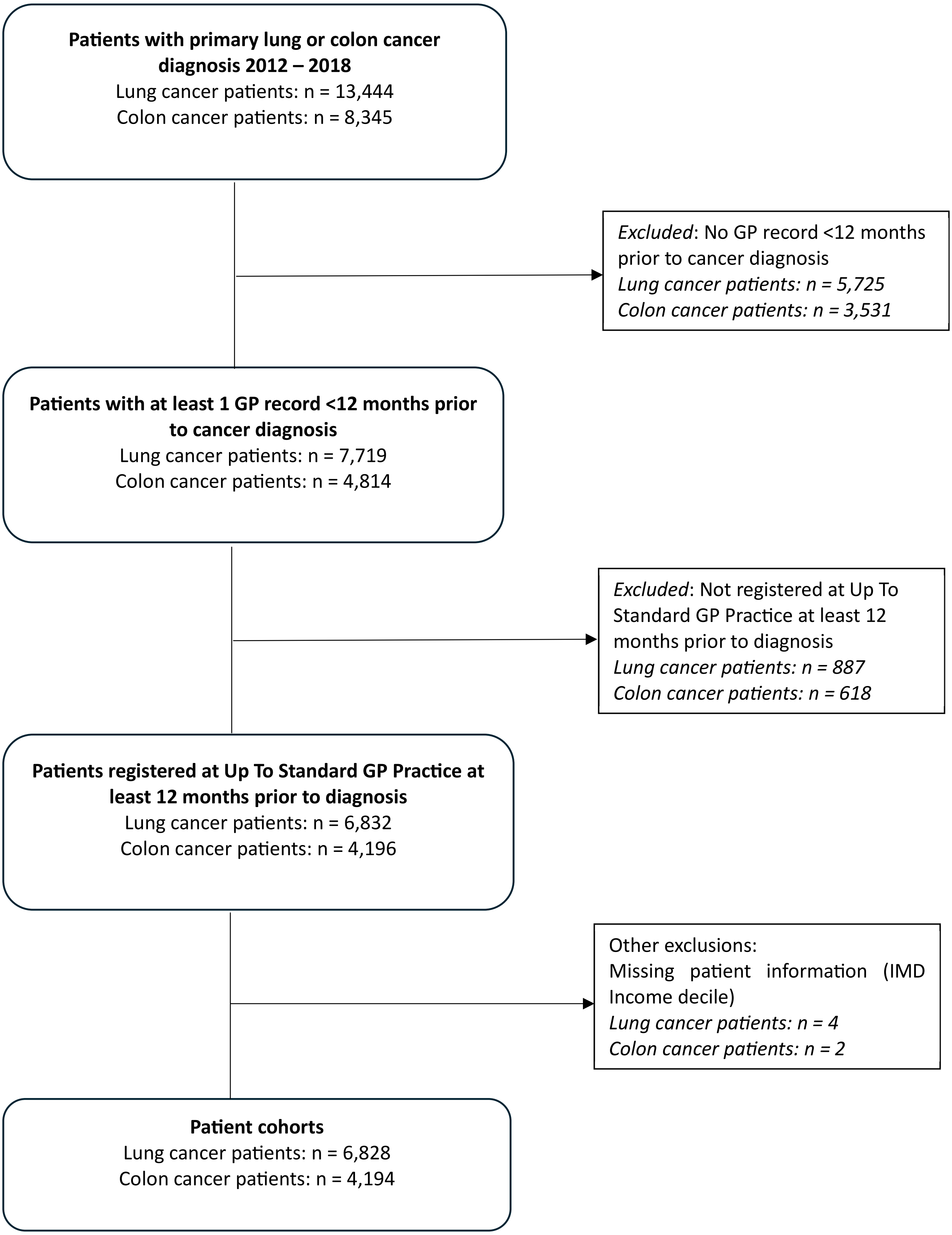

Supplementary Figure 2: Distribution of stage at diagnosis (%) by presence of Anxiety / Depression or Chronic Analgesic Medication. **a** Lung cancer patients (n=6,828) **b**. Colon cancer patients (n=4,194)

a

Lung cancer patients

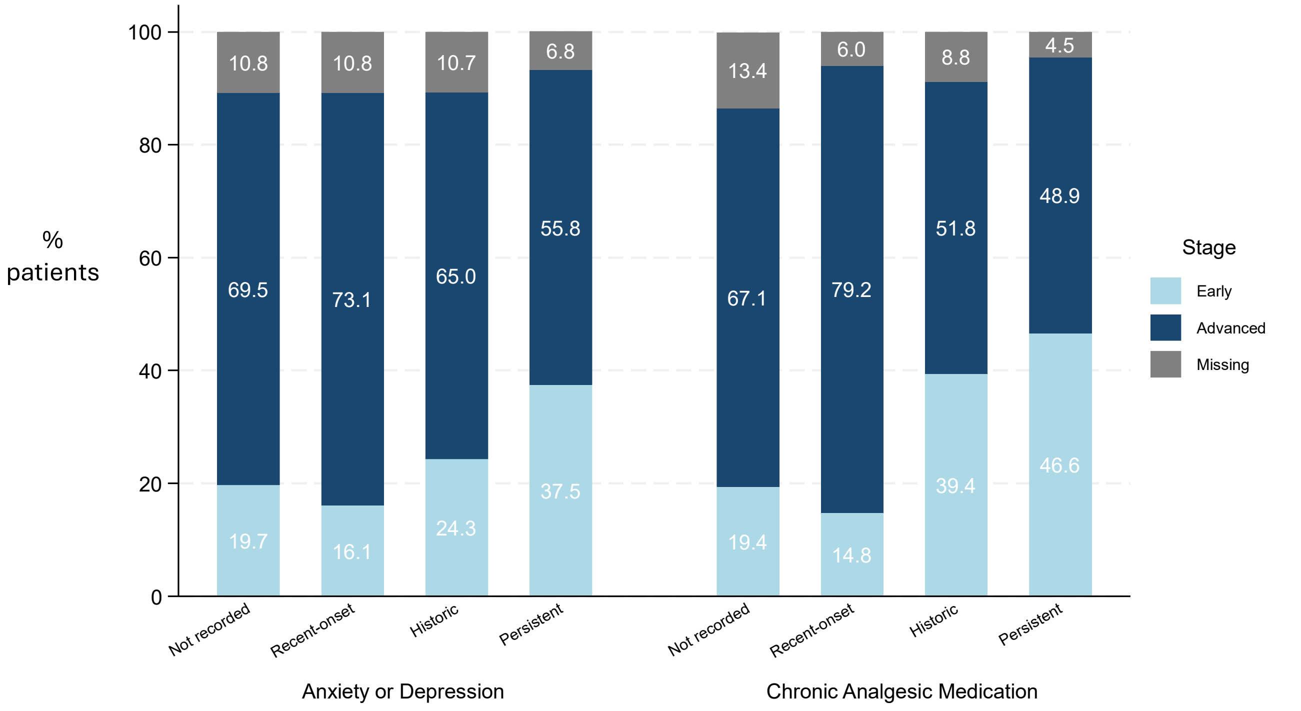

b

Colon cancer patients

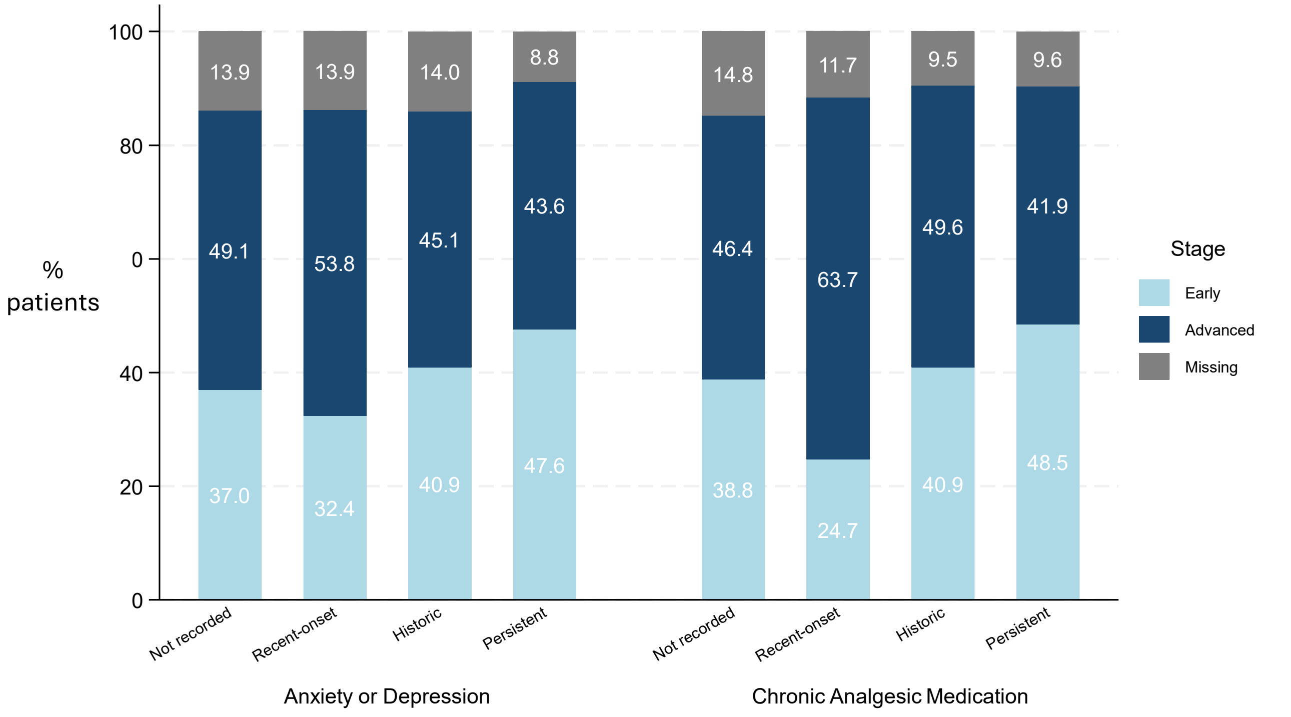

Supplementary Figure 3: Type of drugs prescribed to patients with Chronic Analgesic Medication use, by cancer and by recent-onset or persistent Chronic Analgesic Medication use. **a.** Colon cancer patients (recent-onset use, n=575; persistent use, n=427) **b.** Lung cancer patients (recent-onset use,n=1,859; persistent use, n=600)

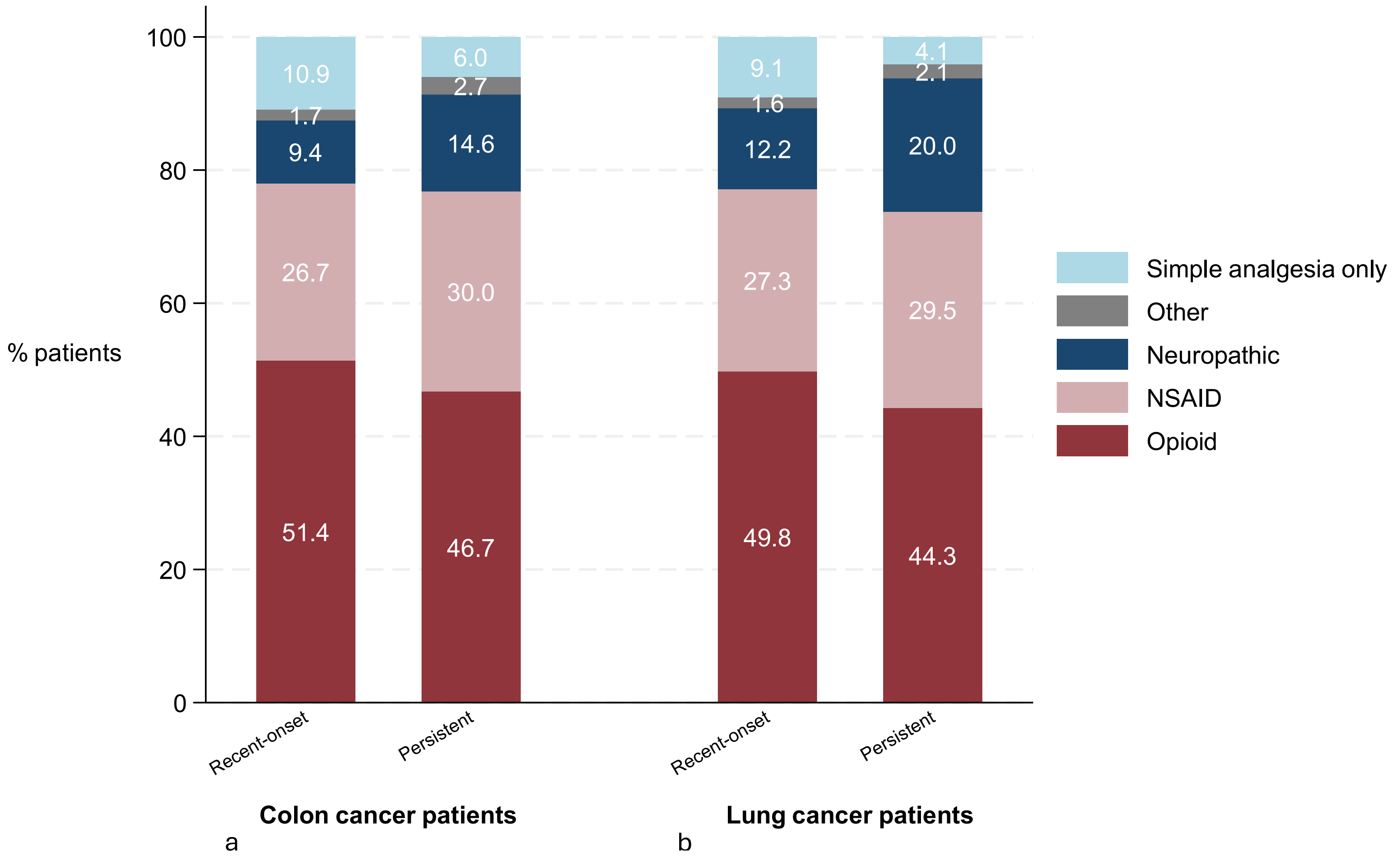

Supplementary Figure 4: Prevalence (% of patients) of Anxiety versus Depression among cancer patients, according to duration. **a** Lung cancer patients (n=6,828) **b**. Colon cancer patients (n=4,194)

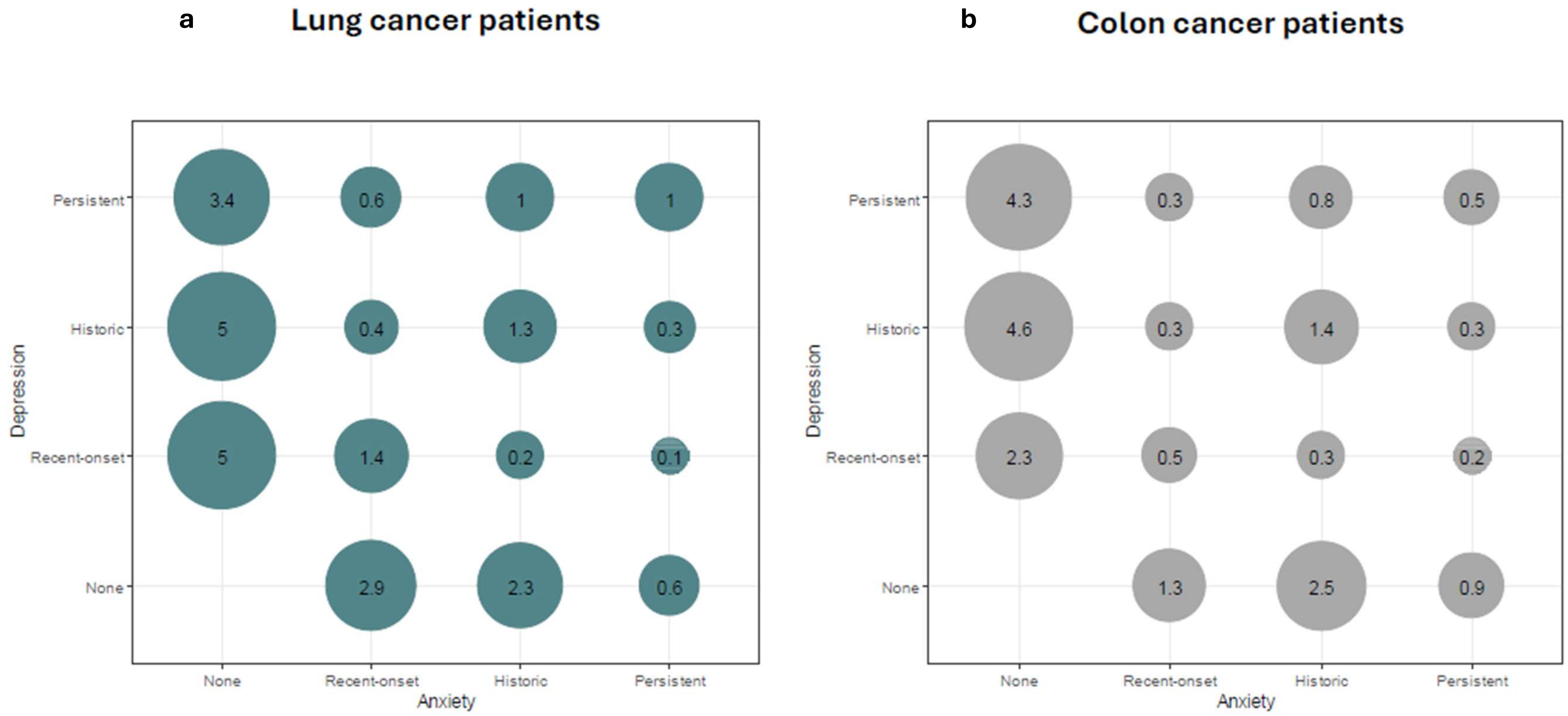

Recent-onset: <12 mths pre-cancer; **Historic:** 12-72 mths pre-cancer; **Persistent:** < 12 mths & 12-72 mths pre-cancer

Supplementary Figure 5: Directed Acyclic Graph showing associations between Chronic Analgesic Medication or Anxiety / Depression, covariates and stage at diagnosis

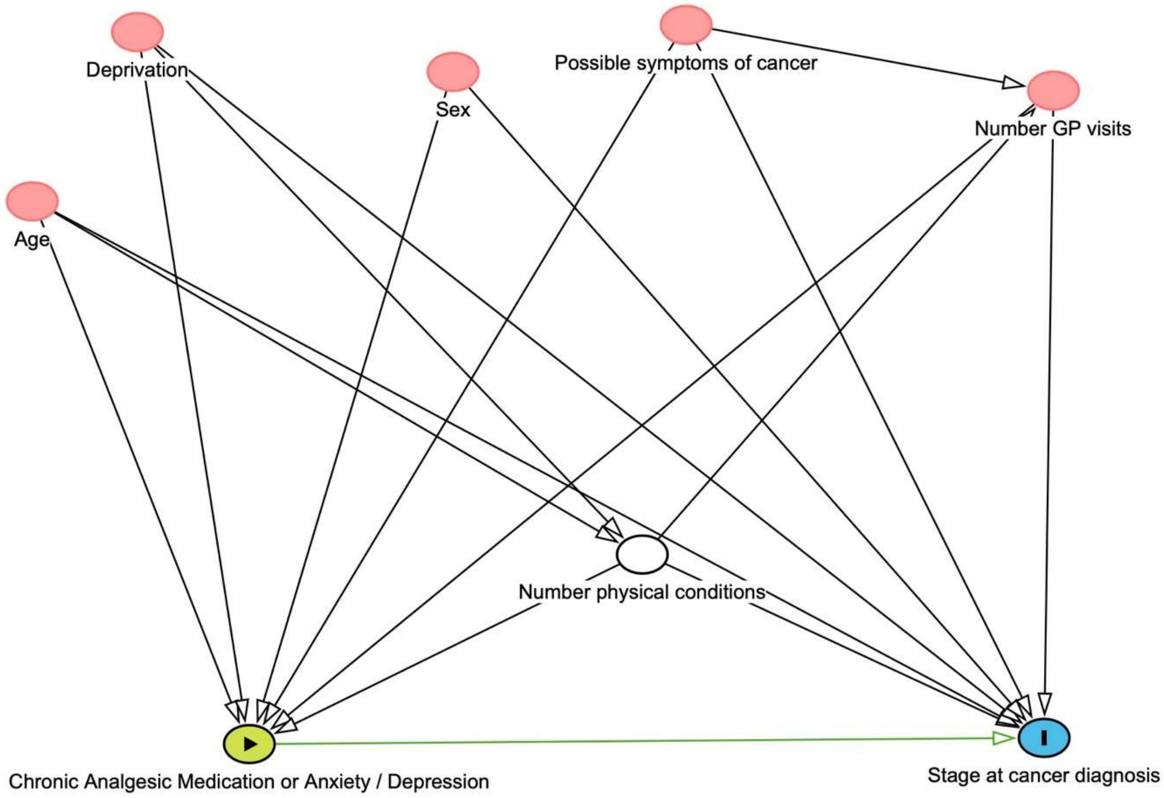

Supplement: Supplementary file 1 — Supplementary Information [file 41467_2025_66334_MOESM1_ESM.pdf]
