# Supplementary material for: Cancer stage at diagnosis by duration of pre-existing chronic analgesic use and anxiety or depression
Source: Nat Commun. 2025 Dec 16;17:7. doi: 10.1038/s41467-025-66334-2 (PMC12764921; doi:10.1038/s41467-025-66334-2)
Supplement: Supplementary file 3 — Description of Additional Supplementary Files [file 41467_2025_66334_MOESM3_ESM.pdf]

<u>**Description of Additional Supplementary Files**</u>

Supplementary Dataset 1: **Prevalence of 35 health conditions among patients diagnosed with lung or colon cancer between 2012 and 2018.** Prevalence calculated as the percentage of all patients with the condition recorded any time up to 72 months pre-cancer (‘Overall prevalence’) and the percentage of all patients with the condition only recorded <12 months pre-cancer (‘Recent’), 12-72 months pre-cancer (‘Historic’) or recorded <12 months and 12-72 months pre-cancer (‘Persistent’).

Supplementary Dataset 2: **Adjusted Odds Ratios (aOR) with 95% CI of diagnosis at stage IV vs stages IIII, compared with results from main analysis (stages III and IV vs stages I and II), estimated using multivariable logistic regression analysis**
